# A comparative analysis and survival analysis of open versus minimally invasive radical antegrade modular pancreatosplenectomy for pancreatic cancer: a systematic review and meta-analysis

**DOI:** 10.3389/fonc.2024.1513520

**Published:** 2025-01-23

**Authors:** Yating Zhou, Fei Xue

**Affiliations:** Kunshan Hospital of Traditional Chinese Medicine, Suzhou, Jiangsu, China

**Keywords:** minimally invasive RAMPS, meta-analysis, open RAMPS, pancreatic cancer, surgical outcomes

## Abstract

**Background:**

Pancreatic ductal adenocarcinoma (PDAC) is a major public health concern, ranking as the fourth leading cause of cancer-related mortality in the United States. Traditional surgical approaches often yield suboptimal outcomes, highlighting the need for innovative surgical strategies. Radical antegrade modular pancreatosplenectomy (RAMPS) has demonstrated improvements in surgical visualization and oncological outcomes. Recently, laparoscopic RAMPS (L-RAMPS) has been introduced as a minimally invasive alternative.

**Objectives:**

This meta-analysis aims to compare the safety and efficacy of open RAMPS (O-RAMPS) versus L-RAMPS, focusing on operative outcomes, minimally invasive outcomes, intra-abdominal outcomes, overall postoperative outcomes, and oncologic outcomes.

**Methods:**

A systematic review and meta-analysis were conducted following PRISMA guidelines. Eligible studies included prospective or retrospective cohort studies and randomized controlled trials comparing L-RAMPS with O-RAMPS. Data were extracted from EMBASE, PubMed, and the Cochrane Library databases through September 16, 2023. The ROBINS-I tool was used to assess the risk of bias. Statistical analyses included odds ratios (OR), risk differences (RD), mean differences (MD), and survival analyses.

**Results:**

Eight studies involving 588 patients were included. O-RAMPS was associated with longer operative times (MD = 39.39 minutes, 95% CI = 22.93 to 55.84) and greater blood loss (MD = -231.84 mL, 95% CI = -312.00 to -151.69). No significant differences were observed in blood transfusion rates, pancreatic fistula rates, delayed gastric emptying, or length of hospital stay. L-RAMPS demonstrated a shorter time to oral feeding (MD = -0.79 days, 95% CI = -1.35 to -0.22). Survival analysis suggested a potentially improved long-term prognosis for L-RAMPS.

**Conclusion:**

L-RAMPS offers advantages over O-RAMPS in terms of reduced blood loss, faster time to oral feeding, and potentially better long-term prognosis. Further research is warranted, particularly regarding the learning curve of L-RAMPS and its broader applicability.

**Systematic review registration:**

https://www.crd.york.ac.uk/prospero, identifier CRD42024498383.

## 1 Introduction

Pancreatic ductal adenocarcinoma (PDAC) is a significant threat to human health, ranking as the fourth leading cause of cancer-related deaths in the U.S (1). A particular challenge is that PDAC affecting the body and tail often progresses without symptoms until late stages. This results in advanced disease and metastasis at the time of diagnosis, reducing the possibility of successful surgical removal (2–6). In general, the standard surgical approach for cancers of the body and tail of the pancreas is open distal pancreatectomy with en-bloc regional lymph node dissection. While conventional distal pancreatectomy follows a left-to-right approach, challenges in visualizing the posterior margin can contribute to a high rate of positive margins, early recurrence, and limited survival (7). Radical antegrade modular pancreatosplenectomy (RAMPS), an advanced surgical approach introduced by Strasberg et al. (7) and Grossman et al (8), has provided improved surgical field visibility of the posterior plane and anatomic landmarks, proper adjustment of the depth of the posterior extent of resection according to the depth to the posterior outline of the tumor, early vascular control, and oncologic benefits. RAMPS emphasizes precise localization of the posterior plane of dissection, increasing the potential for extensive nodal removal and a higher chance of achieving negative margins under the microscope (9).

From a technical standpoint, RAMPS applies the same oncologic principles to left-sided pancreatic tumors that are used for radical resection of pancreatic head tumors. Previous studies have demonstrated the safety and efficacy of RAMPS, leading to the exploration of laparoscopic RAMPS (L-RAMPS) in recent years, especially in high-volume pancreatic centers for selected cases. In L-RAMPS, access to the superior mesenteric artery (SMA) and celiac trunk (CT) is obtained by early division of the pancreatic neck, splenic vein and artery. Retroperitoneal dissection then proceeds along the right side of the SMA and CT to the aorta, and thus medial to lateral, until the specimen is fully mobilized en-bloc with the spleen. Depending on the depth of the posterior plane of dissection, RAMPS may be performed anteriorly (i.e., including the anterior renal fascia) or posteriorly (i.e., removing the left adrenal gland en-bloc) (7, 9, 10). In minimally invasive RAMPS (L-RAMPS), the retroperitoneal dissection is performed in a cranial direction with an early approach to the posterior plane, which does not necessarily require early division of the above structures. Overall, RAMPS offers the advantage of vascular-oriented dissection, allowing for radical dissection of the extrapancreatic nerve plexus, and improves visualization of the posterior plane, potentially reducing the margin positivity rate at this level. The final dissection is said to be identical in O-RAMPS and L-RAMPS, but this is done from a different anatomical approach, raising an important question about the safety and feasibility of L-RAMPS.

This article aims to conduct a systematic review of relevant literature, comparing the effectiveness and safety of two surgical approaches.

## 2 Methods

This work has been reported in line with the PRISMA (Preferred Reporting Items for Systematic Reviews and Meta-Analyses) (11) and AMSTAR (Assessing the methodological quality of systematic reviews) Guidelines (12). To avoid reporting bias and duplication, we pre-registered the study protocol in PROSPERO (https://www.crd.york.ac.uk/prospero) with the registration number CRD42024498383.

A systematic search of the online databases EMBASE (via Elsevier), PubMed, and the Cochrane Library was performed from their inception to June 16, 2024. The search strategy (**Supplementary Data Sheet 1**) was developed in collaboration with a collaborator. Inclusion criteria were: (1) published prospective or retrospective cohort studies or randomized controlled trials (RCTs); (2) studies comparing L-RAMPS with O-RAMPS; (3) studies reporting at least one of the outcomes of interest. Studies were excluded if the publication language was not English or Chinese. Animal studies, reviews, congress abstracts, editorials, letters, commentaries, case reports and case series were also excluded. After an initial joint screening calibration phase, 2 reviewers (Xue and Zhou) performed title and abstract screening. The same reviewers performed full-text screening independently, and disagreements were discussed until consensus was reached. In addition, only studies with Kaplan-Meier (KM) survival curves were included in the KM-based meta-analysis. Two reviewers (Xue and Zhou) independently screened the studies and extracted data according to predefined criteria. The extracted data included general information (first author, year of publication, country, study design, sample size, study period, patient age and sex) and 12 outcomes (operation time, blood loss, blood transfusion, pancreatic fistula, delayed gastric emptying (DGE), length of stay, time between surgery and chemotherapy, time to oral feeding, tumor size, harvested lymph nodes, lymph node positive rate, R0 resection). Pancreatic fistula was defined according to the latest standards of the International Study Group of Pancreatic Surgery (ISGPS) (13). On postoperative day 3 or later, an increase in the amylase level of the drainage fluid to more than three times the upper limit of normal is diagnosed as a Grade A pancreatic fistula (biochemical leak). A Grade B pancreatic fistula is defined by one or more of the following: prolonged drainage (>3 weeks), modification of clinical management, requirement for percutaneous or endoscopic drainage, bleeding requiring interventional angiography, or infection without organ failure. Grade C pancreatic fistula involves surgical intervention, organ failure, or mortality. DGE was defined according to previously proposed criteria (14). Grade A: nasogastric intubation (NGT) lasting >3 postoperative days (POD) or inability to tolerate solid food by POD 7. Grade B: NGT lasting 8–14 days, reinsertion of NGT after POD 7, or prolonged gastric drainage delaying return to solid food. Grade C: NGT lasting >14 days, reinsertion after POD 14, or inability to tolerate solid food by POD 21.Postoperative complications were graded according to the Clavien-Dindo classification of surgical complications (15). An iterative approach, originally developed by Guyot et al. (16) and refined by Wei and Royston (17) and Liu et al. (18), was used to obtain reconstructed patient data (RPD) from the included original studies. This approach has been previously described in detail and its high accuracy and reproducibility has been repeatedly demonstrated (16–18). For this study, a digital software program (DigitizeIt, version 2.5.9; DigitizeIt Services) was used to import the quality data coordinates (time and survival probability) of the published KM curves. The patient data of each included study were reconstructed using the web application Shiny, version 1.2.3.0 (last update: 03/22/2022) (19), which was developed by Liu et al. (18) and integrates the iterative algorithm into a user-friendly web application. Two independent reviewers (Xue and Zhou) assessed the RPD and KM curves of each study cohort, and the accuracy was calculated by visual inspection. The RPD from each study was pooled for further analysis. RPD from studies reporting PDAC survival after RAMPS were pooled to obtain the long-term outcome. Researches missing certain information were excluded from the analysis. Risk of bias was assessed, where applicable, using the Risk of Bias in Non-randomized Studies of Interventions (ROBINS-I) tool (20). The ROBINS-I tool assesses 7 domains (confounding, selection of participants into the study, classification of interventions, departures from the intended interventions, missing data, measurement of outcomes, and selection of the reported outcome) through which bias may be introduced into nonrandomized studies. Two reviewers (Xue and Zhou) independently assessed the risk of bias for each included study using a standardized form. Disagreements were discussed until consensus was reached.

Meta-analysis was performed according to the Cochrane Reviewer’s Handbook. Statistical analyses were conducted using Review Manager (RevMan) 5.3 (The Cochrane Collaboration, Nordic Cochrane Centre, Copenhagen, Denmark) and R (version 4.3.2). Results were presented as odds ratio (OR) or risk difference (RD) with 95% confidence interval (CI) for dichotomous data, and mean difference (MD) with 95% CI for continuous data. The weighted mean was also calculated as a reference for outcomes presented as continuous data. Heterogeneity between studies was assessed using the chi-squared test (P < 0.10 indicated statistically significant heterogeneity) and the I^2^ statistic (I^2^ > 50% indicated significant heterogeneity). If no significant heterogeneity was found, a fixed-effects model was used. Otherwise, a random-effects model was applied. Publication bias for outcomes with more than 10 included studies was evaluated using funnel plots, with a symmetric distribution indicating no publication bias. Pooled analyses were visualized using forest plots, and statistical significance was considered at P < 0.05. For studies that did not provide mean and standard deviation (SD), the sample mean and SD were calculated using the following website and relevant data: [Conversion of median to mean](www.math.hkbu.edu.hk/~tongt/papers/median2mean.html).

## 3 Results

**Figure 1:**
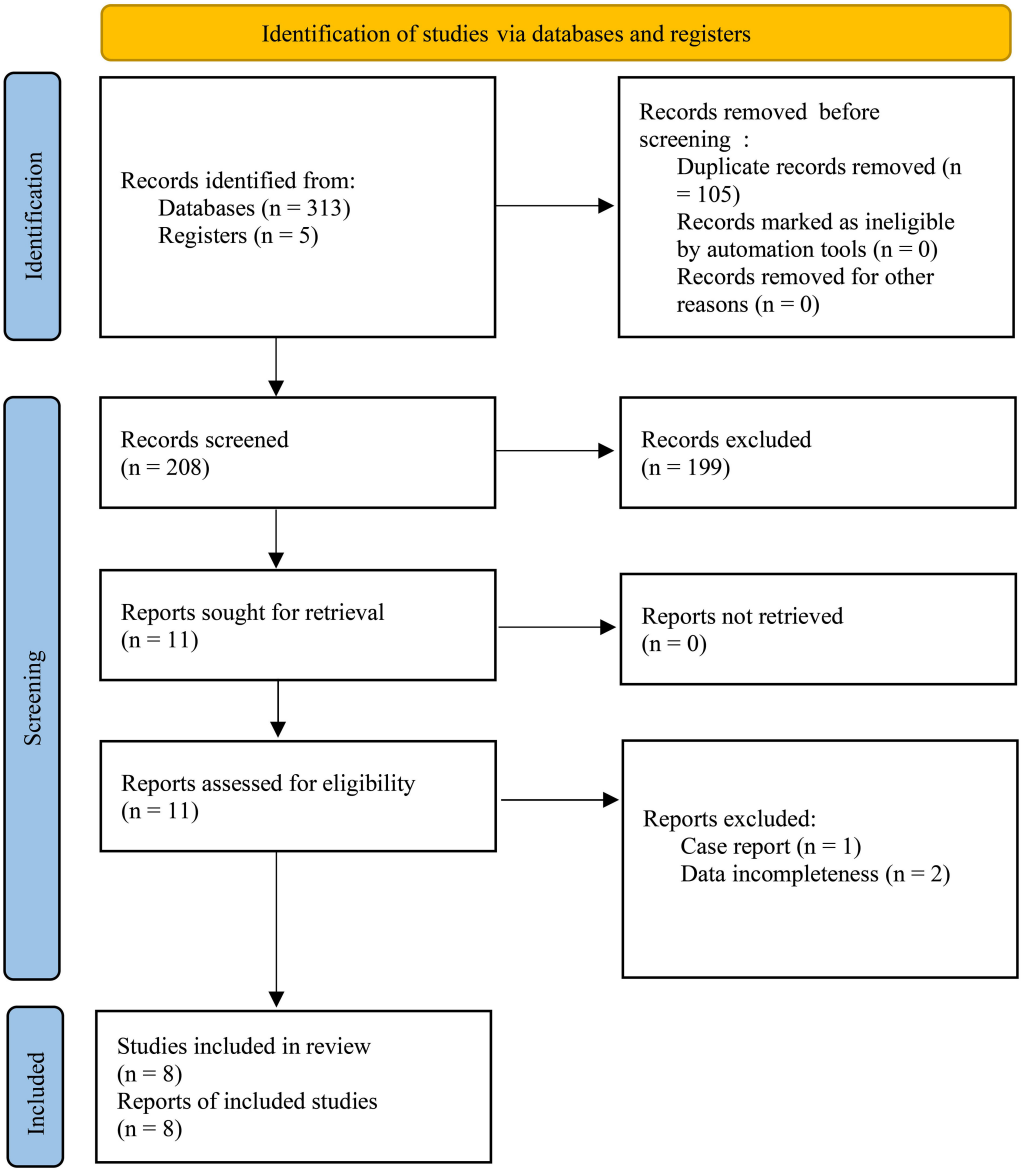
PRISMA Flow Diagram of Study Selection Process. A total of 318 records were identified from databases and registers, with 105 duplicate records removed before screening. After screening 208 records, 199 were excluded. Eleven reports were assessed for eligibility, and 3 reports were excluded due to case report and incomplete data, resulting in 8 studies being included in the final review.

A total of 318 records were identified through database searches and registries. After removing duplicates (n = 105), the remaining 208 records were screened for titles and abstracts. Of the resulting 11 full-text articles, 8 studies (21–28) with 588 patients were included in the final qualitative and quantitative analysis (**Figure 1**). A total of 6 studies with 387 patients were used for the KM-based meta-analysis. **Supplementary Figure 1** outlines the study selection process.

The brief characteristics of the included studies are presented in **Table 1**, and a detailed summary of the included studies is presented in **Supplementary Data Sheet 2**. Studies were conducted in 8 countries: Europe (n = 2) and Asia (n = 6), with sample sizes ranging from 23 to 178. According to the results of the risk of bias assessment for the studies included in the meta-analysis, 4 studies were considered to be at low risk of bias, 3 studies were considered to be at moderate risk of bias, and 1 study was considered to be at high risk of bias (**Supplementary Figures 1**, **2**).

**Table 1:** Brief summary of studies included.

Source	Region	Study periods	Study design	No. of patients			Age, mean (SD) or median (IQR), year		Gender, male/female		KM-based survival meta-analysis
				Overall	L-RAMPS	O-RAMPS	L-RAMPS	O-RAMPS	L-RAMPS	O-RAMPS	
Ricci et al.	Italy	2011-2021	RCS	178	93	85	68.6 ± 8.8	67.6 ± 11.4	46/47	38/47	No
Hirashita et al.	Japan	2007-2019	RCS	50	19	31	73.8 ± 8.1	71.4 ± 9.1	13/6	20/11	Yes
Huang et al.	China	2014-2018	RCS	51	20	31	67.2 ± 8.4	66.9 ± 9.1	10/10	15/16	Yes
Kawabata et al.	Japan	2013-2019	RCS	30	15	15	74 (52–85)	76 (55–86)	9/6	9/6	Yes
Rosso et al.	Italy	2014-2018	RCS	23	17	6	71	75	8/9	4/2	No
Zhang et al.	China	2012-2018	RCS	48	25	23	64.72 ± 9.11	63.26 ± 8.74	17/8	11/12	Yes
Sato et al.	Japan	2016-2021	RCS	118	43	75	69 (35–84)	70 (37–89)	18/25	49/26	Yes
Lee et al.	South Korea	1996-2012	RCS	90	12	78	63.3 ± 9.9	51.2 ± 9.9	7/5	47/31	Yes

### 3.1 Operation time

**Figure 2:**
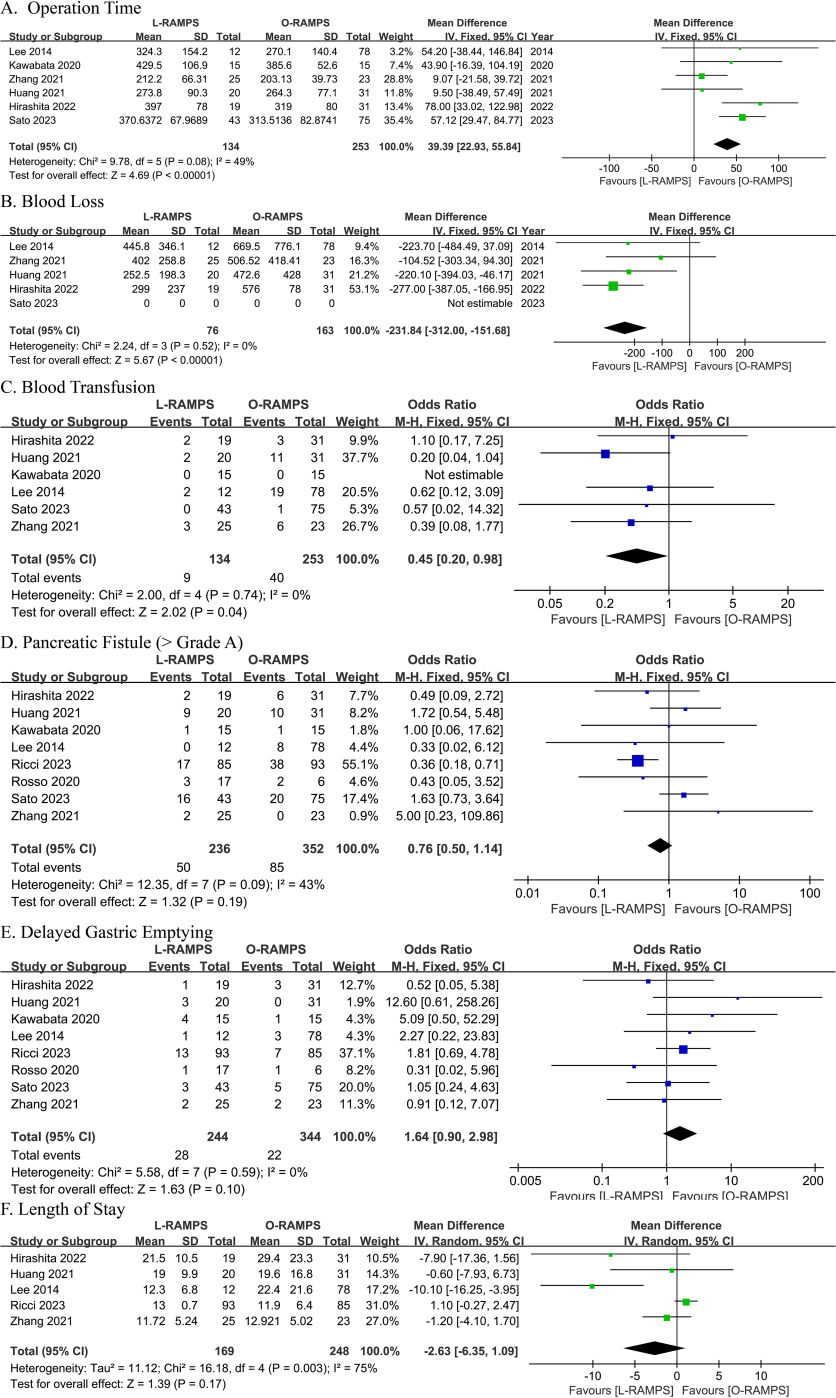
Comparative Analysis of Outcomes Between L-RAMPS and O-RAMPS This figure presents a comparative analysis of various clinical outcomes between Laparoscopic Radical Antegrade Modular Pancreatosplenectomy (L-RAMPS) and Open Radical Antegrade Modular Pancreatosplenectomy (O-RAMPS) for pancreatic cancer. **(A)** Operation Time: Six studies indicated a significantly shorter operation time for O-RAMPS (mean difference = 39.39 minutes, 95% CI = 22.93 to 55.84, P < 0.00001) with no significant heterogeneity (P = 0.08, I² = 49%). **(B)** Blood Loss: Five studies showed significantly less blood loss in the L-RAMPS group (mean difference = -231.84 mL, 95% CI = -312.00 to -151.68, P < 0.00001) with no significant heterogeneity (P = 0.52, I² = 0%). **(C)** Blood Transfusion: Six studies indicated a significantly lower blood transfusion rate for L-RAMPS (odds ratio = 0.45, 95% CI = 0.20 to 0.98, p = 0.04) with no significant heterogeneity (P = 0.74, I² = 0%). **(D)** Pancreatic Fistula (Grade > A): Seven studies showed no significant difference in pancreatic fistula rates (odds ratio = 0.76, 95% CI = 0.50 to 1.14, p = 0.19) with no significant heterogeneity (P = 0.09, I² = 43%). **(E)** DGE: Seven studies showed no significant difference in DGE rates (odds ratio = 1.64, 95% CI = 0.90 to 2.98, p = 0.10) with no significant heterogeneity (P = 0.59, I² = 0%). **(F)** Length of Stay: Five studies showed no significant difference in hospital stay duration (mean difference = -2.63 days, 95% CI = -6.35 to 1.09, p = 0.17) with significant heterogeneity (P = 0.003, I² = 75%).

Six studies provided information on operation time. Among them, two suggested that operative time was longer with O-RAMPS, while the remaining four studies showed no significant difference between L-RAMPS and O-RAMPS. No significant heterogeneity was observed (P=0.08, I²=49%). Using a fixed-effects model, the analysis showed that the operating time for L-RAMPS was longer than for O-RAMPS (mean difference = 39.39 minutes, 95% CI = 22.93-55.84, P<0.05) (**Figure 2A**).

### 3.2 Blood loss

The blood loss analysis included data from four studies. Among these studies, two found no statistically significant difference between L-RAMPS and O-RAMPS in blood loss, while two suggested a trend toward reduced blood loss in the L-RAMPS group. Notably, no significant heterogeneity was observed among these studies (P = 0.52, I² = 0%). Using a fixed-effects model, the aggregated results showed a significant reduction in blood loss with L-RAMPS compared to O-RAMPS (mean difference = -231.84 mL, 95% CI = -312.00 to -151.69, P < 0.05) (**Figure 2B**).

### 3.3 Blood transfusion

Data on blood transfusion were contributed by six studies. None of these studies showed a statistically significant difference between L-RAMPS and O-RAMPS (P = 0.05, I² = 0%). However, using a fixed-effects model, the combined results indicated a higher rate of blood transfusion in the L-RAMPS group compared with the O-RAMPS group (odds ratio = 0.45, 95% CI = 0.20 - 0.98, p = 0.04) (**Figure 2C**).

### 3.4 Pancreatic fistula (grade B and C)

Eight studies were included in the analysis of pancreatic fistula rates (Grade > A). Seven of these studies reported no significant difference in pancreatic fistula rates between the L-RAMPS and O-RAMPS groups. However, one study suggested a higher incidence of pancreatic fistula in the O-RAMPS group. Notably, there was no significant heterogeneity among the studies (P = 0.09, I² = 43%). Using a fixed-effects model, the analysis showed no statistically significant difference in pancreatic fistula rates between L-RAMPS and O-RAMPS (odds ratio = 0.76, 95% CI = 0.50 to 1.14, p = 0.19) (**Figure 2D**).

### 3.5 Delayed gastric emptying

Eight studies were conducted to compare gastric emptying time between the L-RAMPS and O-RAMPS groups. None of the studies showed a significant difference in gastric emptying delay between patients in the L-RAMPS and O-RAMPS groups. In addition, no significant heterogeneity in gastric emptying delay was observed across the eight studies (P = 0.59, I^2^ = 0%). Using a fixed-effects model, the analysis showed that the rate of DGE in the L-RAMPS group was similar to that in the O-RAMPS group (odds ratio = 1.64, 95% CI = 0.90 to 2.98, p = 0.10) (**Figure 2E**).

### 3.6 Length of stay

Length of stay data were available from five studies. Among these, four studies suggested no significant difference between L-RAMPS and O-RAMPS, while one study indicated a shorter length of stay in the L-RAMPS group. Significant heterogeneity was observed (P = 0.003, I² = 75%). Using a random-effects model, the analysis showed no significant difference in length of stay between the L-RAMPS and O-RAMPS groups (mean difference = -2.63 days, 95% CI = -6.35 to 1.09, p = 0.17) (**Figure 2F**).

### 3.7 Time between surgery and chemotherapy

**Figure 3:**
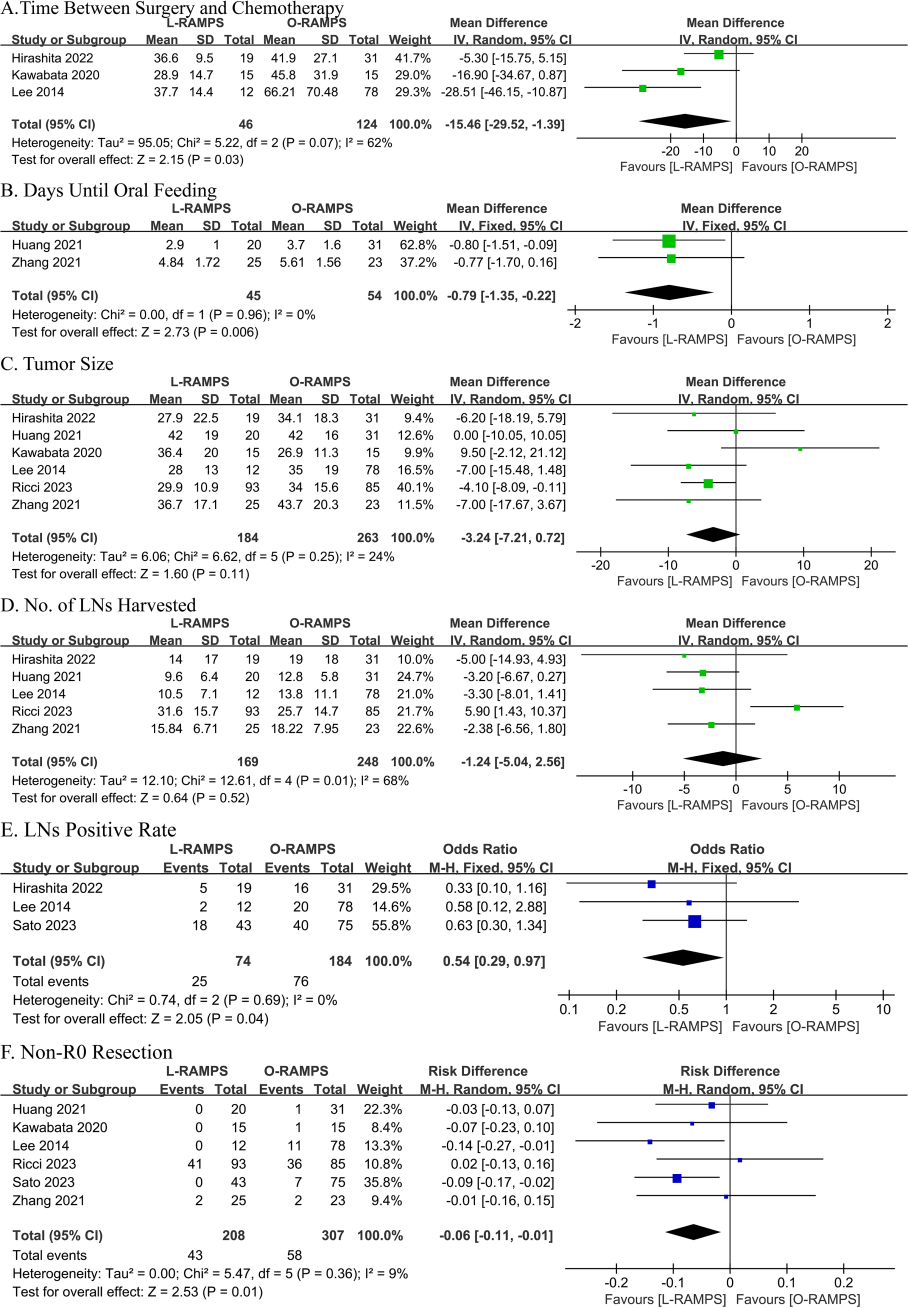
Comparative Analysis of Outcomes Between L-RAMPS and O-RAMPS. This figure presents a comparative analysis of various clinical outcomes between Laparoscopic Radical Antegrade Modular Pancreatosplenectomy (L-RAMPS) and Open Radical Antegrade Modular Pancreatosplenectomy (O-RAMPS) for pancreatic cancer. **(A)** Time Between Surgery and Chemotherapy: Three studies indicated that the time between surgery and chemotherapy was significantly shorter for L-RAMPS (mean difference = -15.46 days, 95% CI = -29.52 to -1.39, p = 0.03) with significant heterogeneity (P = 0.07, I² = 62%). **(B)** Days Until Oral Feeding: Two studies showed a significantly shorter time to oral feeding in the L-RAMPS group (mean difference = -0.79 days, 95% CI = -1.35 to -0.22, p = 0.006) with no significant heterogeneity (P = 0.96, I² = 0%). **(C)** Tumor Size: Five studies indicated no significant difference in tumor size between L-RAMPS and O-RAMPS (mean difference = -3.24 mm, 95% CI = -7.21 to 0.72, p = 0.11) with no significant heterogeneity (P = 0.25, I² = 24%). **(D)** Number of Lymph Nodes Harvested: Five studies showed no significant difference in the number of lymph nodes harvested between the two groups (mean difference = -1.24, 95% CI = -5.04 to 2.56, p = 0.52) with significant heterogeneity (P = 0.01, I² = 68%). **(E)** Lymph Node Positive Rate: Three studies indicated a significantly lower lymph node positive rate in the L-RAMPS group (odds ratio = 0.54, 95% CI = 0.29 to 0.97, p = 0.04) with no significant heterogeneity (P = 0.69, I² = 0%). **(F)** Non-R0 Resection: Six studies showed a higher non-R0 resection rate in the O-RAMPS group (risk difference = -0.06, 95% CI = -0.11 to -0.01, p = 0.01) with no significant heterogeneity (P = 0.36, I² = 9%).

Three studies reported the time between surgery and chemotherapy. Two of these studies showed no significant difference between the L-RAMPS and O-RAMPS groups, while one study indicated a shorter time between surgery and chemotherapy in the L-RAMPS group. No significant heterogeneity was observed (P = 0.07, I² = 62%). Using a fixed-effects model, the analysis showed a shorter time between surgery and chemotherapy in the L-RAMPS group (mean difference = -15.46 days, 95% CI = -29.52 to -1.39, p = 0.03) (**Figure 3A**).

### 3.8 Days to oral feeding

Data on days to oral feeding were reported in two studies. One study showed no significant difference between L-RAMPS and O-RAMPS, while the other study showed a significantly shorter time to oral feeding in the L-RAMPS group. Notably, no significant heterogeneity was observed (P = 0.96, I² = 0%). Using a fixed-effects model, the analysis showed that the days to oral feeding were significantly shorter in L-RAMPS patients (mean difference = -0.79 days, 95% CI = -1.35 to -0.22, p = 0.006) (**Figure 3B**).

### 3.9 Size of tumor

Six studies reported tumor size. Of these, five studies suggested no significant difference between L-RAMPS and O-RAMPS, while one study indicated that tumors were larger in the O-RAMPS group. Notably, no significant heterogeneity was observed (P = 0.25, I² = 24%). Using a random-effects model, the analysis showed no significant difference in tumor size between L-RAMPS and O-RAMPS patients (mean difference = -3.24 mm, 95% CI = -7.21 to 0.72, p = 0.11) (**Figure 3C**).

### 3.10 Lymph nodes harvested

Five studies provided data on the number of lymph nodes harvested. Among these, four studies suggested no significant difference between L-RAMPS and O-RAMPS, while one study indicated that more lymph nodes were harvested in O-RAMPS patients compared to L-RAMPS patients. Notably, significant heterogeneity was observed (P = 0.01, I² = 68%). Using a random-effects model, the analysis showed no significant difference in the number of lymph nodes harvested between the two groups (mean difference = -1.24, 95% CI = -5.04 to 2.56, p = 0.52) (**Figure 3D**).

### 3.11 Lymph node positive rate

Three studies provided data on the lymph node positive rate. None of these studies reported a significant difference in the lymph node positive rate between L-RAMPS and O-RAMPS. No significant heterogeneity was observed (P = 0.69, I² = 0%). Using a fixed-effects model, the analysis showed a lower lymph node positive rate associated with L-RAMPS (mean difference = 0.54, 95% CI = 0.29 to 0.97, p = 0.04) (**Figure 3E**).

### 3.12 Non-R0 resection

Six studies provided data on R0 resection. Among these, four studies suggested no significant difference between L-RAMPS and O-RAMPS, while two studies indicated a higher non-R0 resection rate in O-RAMPS patients. Notably, no significant heterogeneity was observed (P = 0.36, I² = 9%). Using a random-effects model, the analysis showed that the non-R0 resection rate was higher in O-RAMPS (risk difference = -0.06, 95% CI = -0.11 to -0.01, p = 0.01) (**Figure 3F**).

### 3.13 KM-based meta-analysis

**Figure 4:**
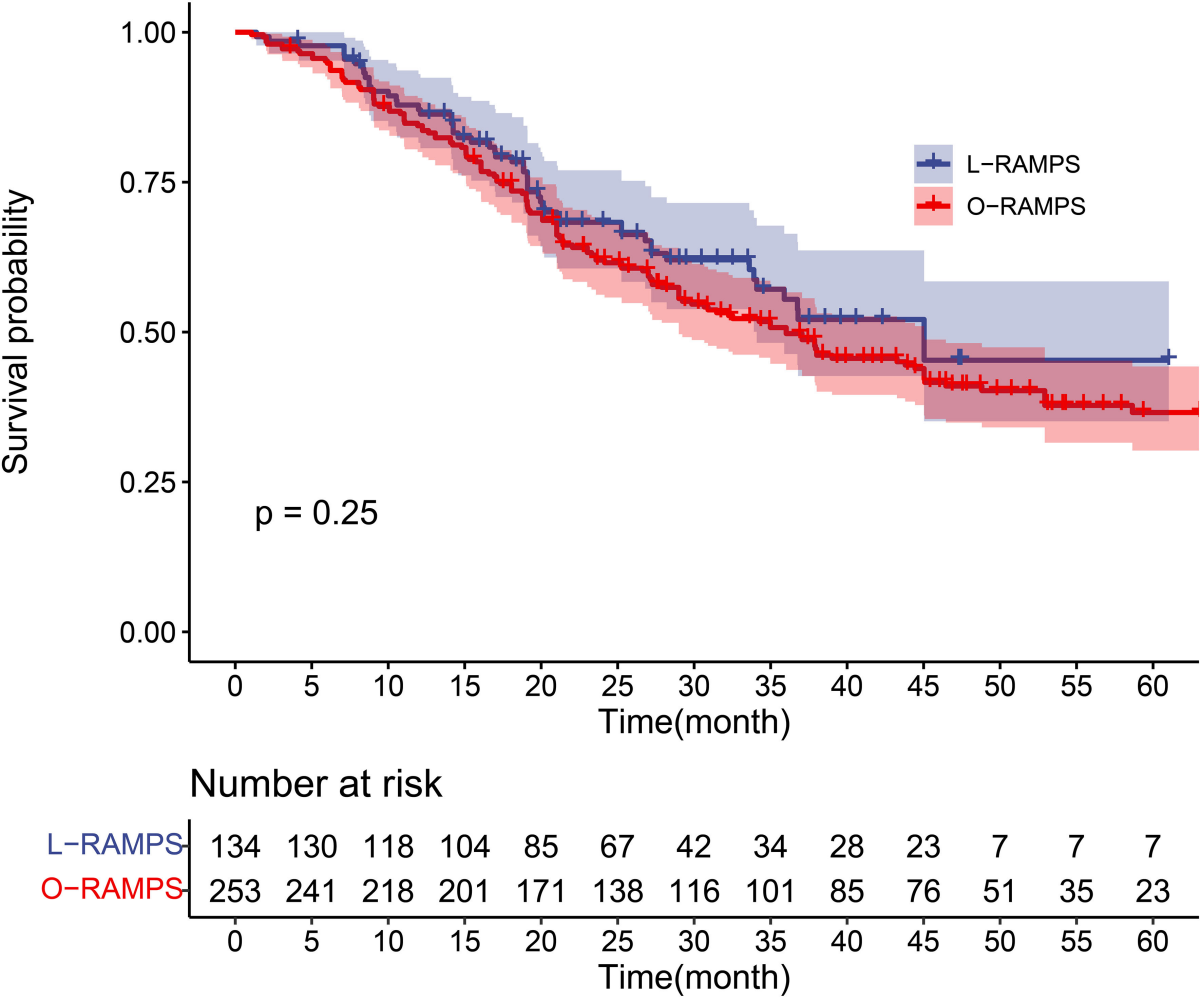
Kaplan-Meier survival curve for PDAC patients undergoing RAMPS. The survival curves of the L-RAMPS group and O-RAMPS group were compared using the log-rank test, and the p-value was 0.25.

A total of six studies involving 384 patients reported the long-term overall survival of individuals who survived PDAC located in the tail of the pancreas following RAMPS procedures. During a cumulative follow-up of four years, 196 deaths were recorded. The mean (SD) survival time was 25.0 (14.6) months, with a median survival time of 37.1 months (IQR, 14.2-35.2 months). Survival rates at the first, second, third, and fourth years were 85.3%, 64.1%, 52.4%, and 42.5%, respectively. Specifically looking at L-RAMPS, the one-year, two-year, and three-year survival rates were 87.1%, 68.3%, and 55.5%, respectively. For O-RAMPS, the survival rates at one, two, three, and four years were 84.8%, 62.0%, 50.7%, and 41.0%, respectively (**Table 2**). Overall, compared to patients who underwent O-RAMPS, those who underwent L-RAMPS had similar survival times (P=0.25) (**Figure 4**).

**Table 2:** Pooled survival data based on reconstructed patient data.

	Survival of PDAC after RAMPS		
	Overall	L-RAMPS	O-RAMPS
No. of patients	384	134	253
Observation time, person-months	11568	3624.9	7943.1
Deaths, No.	196	55	141
Survival time, m			
Mean (SD)	25.0 (14.6)	21.8 (12.0)	27.0 (15.6)
Median (IQR)	37.1 (14.2-35.2)	36.8 (13.8-29.4)	36.0 (15.1-38.2)
Maximum follow-up, m	58.6	45	58.6
Survival rate, %(95%CI)			
1-y Follow-up	85.3 (81.9-89.0)	87.1 (81.6-93.0)	84.4 (80.0-89.0)
2-y Follow-up	64.1 (59.4-69.2)	68.3 (60.6-77.0)	62.0 (56.2-68.4)
3-y Follow-up	52.4 (47.3-58.1)	55.5 (46.3-66.4)	50.7 (44.7-57.6)
4-y Follow-up	42.5 (37.2-48.6)	NA	41.0 (34.9-48.2)
At maximum follow-up	38.6 (32.9-45.2)	45.3 (35.1-58.4)	36.6 (30.2-44.3)

## 4 Discussion

In this review, we included both RCTs and non-randomized studies of interventions (NRSI). The rationale for including RCTs is that they are considered the gold standard for evaluating the efficacy of interventions due to their ability to minimize bias through randomization. However, we also included NRSI to capture a broader range of evidence and real-world effectiveness of the interventions, as RCTs alone may not provide a comprehensive understanding of the intervention’s impact in diverse and pragmatic settings. Including both types of studies allows for a more thorough and nuanced analysis of the available evidence.

PDAC represents a significant clinical challenge, with only 20% of patients diagnosed at a respectable stage (29, 30). Surgical intervention, while potentially curative, is constrained, particularly in advanced cases affecting the body and tail of the pancreas, resulting in a high recurrence rate and poor prognosis (31–33). Optimal surgical outcomes in PDAC depend on achieving an R0 margin and performing radical N1 lymph node resection (4, 33–36). Conventional distal pancreatosplenectomy often falls short due to a significant margin positivity rate and inadequate lymph node resection. RAMPS, introduced by Strasberg et al. in 2003, stands out as a pivotal advancement. Aimed at improving R0 resection rates and lymph node harvest, RAMPS has been supported by retrospective studies conducted by Park et al., Trottman et al., and Abe et al. (37–39) Its utilization in left-sided PDAC cases is growing among medical centers. Ongoing enhancements in laparoscopic techniques, especially laparoscopic distal pancreatectomy, have exhibited potential in decreasing complications and shortening hospital stays for benign and borderline lesions (40–42). While debate persists regarding the utilization of L-RAMPS for PDAC, the scarcity of published studies emphasizes the necessity for additional research (43–46). Comparative analyses reveal the advantages of laparoscopic surgery over open surgery, including reduced blood loss and shorter hospital stays (40, 42, 47, 48). However, the absence of published randomized trials necessitates cautious interpretation and contributes to the ongoing debate concerning the optimal approach to RAMPS. Our meta-analysis indicates that the L-RAMPS surgical procedure entails a significantly longer operative time compared to the O-RAMPS surgical group. Considering the intricacies of laparoscopic surgery and the characteristics of the learning curve in its initial stages, this finding is understandable. The L-RAMPS procedure remains a novel technique, and encountering a learning curve is highly probable. Further investigation is warranted on how to effectively manage the learning curve associated with L-RAMPS. In related studies on laparoscopic pancreaticoduodenectomy (LPD), varying conclusions have been drawn by different investigators. However, most suggest a significant decrease in operative time after performing more than 30 procedures (47, 48). Given the incorporation of studies reporting L-RAMPS with 12 to 25 cases, it is anticipated that with further refinement of surgical techniques, the operative time for L-RAMPS will likely continue to decrease. Regarding surgical bleeding, the estimated blood loss and transfusion rate in the L-RAMPS group are significantly lower than those in the O-RAMPS group, consistent with findings from other laparoscopic surgery studies (49–51). The reduced bleeding may be due to earlier hemostasis of the splenic artery and the magnifying effect of laparoscopy and CO_2_ pressure in laparoscopy. The lower blood loss and transfusion rates are beneficial for postoperative recovery. Pancreatic fistula, often considered the “bane” of complications after pancreatic surgery, has a profound impact on prognosis. According to the 2016 revised International Study Group for Pancreatic Surgery (ISGPS) classification of pancreatic fistula (13), grade A pancreatic fistula is considered a biochemical fistula that does not affect prognosis and does not require a change in treatment strategy. This study focuses on the incidence rates of grade B and C pancreatic fistula and shows no significant difference between the L-RAMPS and O-RAMPS groups (p > 0.05). Surprisingly, both groups exhibit no statistically significant differences in the length of hospital stay and DGE, suggesting that the advantages of minimally invasive surgery are not fully realized. Regarding the hospital length of stay, the L-RAMPS group demonstrates a shorter duration, albeit without statistical significance. The limited experience with L-RAMPS may contribute to a more conservative approach to patient discharge, thereby explaining the comparable length of hospital stay. With increasing experience in perioperative management, it is expected that the length of stay for L-RAMPS patients will continue to decrease. The occurrence rate of DGE shows no statistically significant difference between the two groups, which contrasts with findings from previous studies (49–51), and the possible reasons warrant further investigation. Although there is no improvement in the rate of DGE, L-RAMPS patients have a shorter postoperative fasting time compared to O-RAMPS patients, which is consistent with previous research. This facilitates a faster return to a normal diet and daily life for patients. Additionally, the time from surgery to the start of chemotherapy is shorter in the L-RAMPS group, potentially benefiting long-term oncologic outcomes. In terms of pathology results, contrary to expectations, there is no significant difference in tumor diameter between the two groups. The analysis indicates that patients who underwent open surgery tended to have larger tumor diameters, but this difference is not statistically significant. As L-RAMPS is still in its early stages, surgeons may prefer to select patients with smaller tumor diameters. The difference in study results may indicate some selectivity and bias in subject selection. The number of lymph nodes retrieved shows no difference between the two groups, which is consistent with previous research. L-RAMPS patients have a lower margin positivity rate, and both lymph node retrieval and margin positivity are effective indicators of oncologic prognosis. The advantages of laparoscopic surgery may be attributed to the increased flexibility of view and the magnifying effect of the laparoscope. The results of the analysis may suggest that L-RAMPS may have a potentially better long-term prognosis than O-RAMPS. This study performed survival analysis based on RPD(reconstructed personal data), which is considered the gold standard for statistical analysis and survival analysis (52). The IPD-based meta-analysis shows a slightly higher survival rate for l-RAMPS compared to o-RAMPS, although without statistical significance (p = 0.25). This may be related to the lower margin positivity rate in l-RAMPS.

This study has certain limitations. Firstly, the sample size in terms of literature inclusion is relatively small, preventing us from conducting sensitivity analysis and gaining a deeper understanding of the heterogeneity within the literature. Secondly, to obtain sufficient data, we included some studies with a higher risk of bias in the analysis. Currently, these limitations cannot be effectively addressed. Further studies are needed to obtain more information about the advantages and disadvantages of the two procedures.

## 5 Conclusion

In summary, compared to O-RAMPS, L-RAMPS demonstrates good safety and efficacy. However, further data are required to support broader implementation. It is important to note that the current centers performing L-RAMPS are high-volume centers, and the current research results may only be applicable to such centers, cautioning against blind generalization to all institutions.

## Data Availability

The original contributions presented in the study are included in the article/**Supplementary Material**. Further inquiries can be directed to the corresponding author.

## Supplementary material

Supplementary Figure 1 Risk of bias assessment across studies. Studies such as Rosso et al., 2020 (26) exhibit a serious risk in D3 and overall bias, while Huang et al., 2021 (22), Zhang et al., 2021 (28), and others show consistently low risk across all domains. Lee et al., 2014 (24) and Kawabata et al., 2020 (23) show moderate risk in D1 and D6, while Hirashita et al., 2022 (21) has a moderate risk in D1 and D2. Two studies (Rosso et al., 2020 (26), Kawabata et al., 2020 (23)) have uncertainties regarding missing data (D5), indicated by blue question marks. The overall bias summary highlights both high- and low-risk studies, providing an overview of the potential limitations in the included literature.

Supplementary Figure 2 Risk of bias summary across bias domains.
