# Supplementary figures and images for: A comparative analysis and survival analysis of open versus minimally invasive radical antegrade modular pancreatosplenectomy for pancreatic cancer: a systematic review and meta-analysis
Source: Front Oncol. 2025 Jan 23;14:1513520. doi: 10.3389/fonc.2024.1513520 (PMC11798776; doi:10.3389/fonc.2024.1513520)

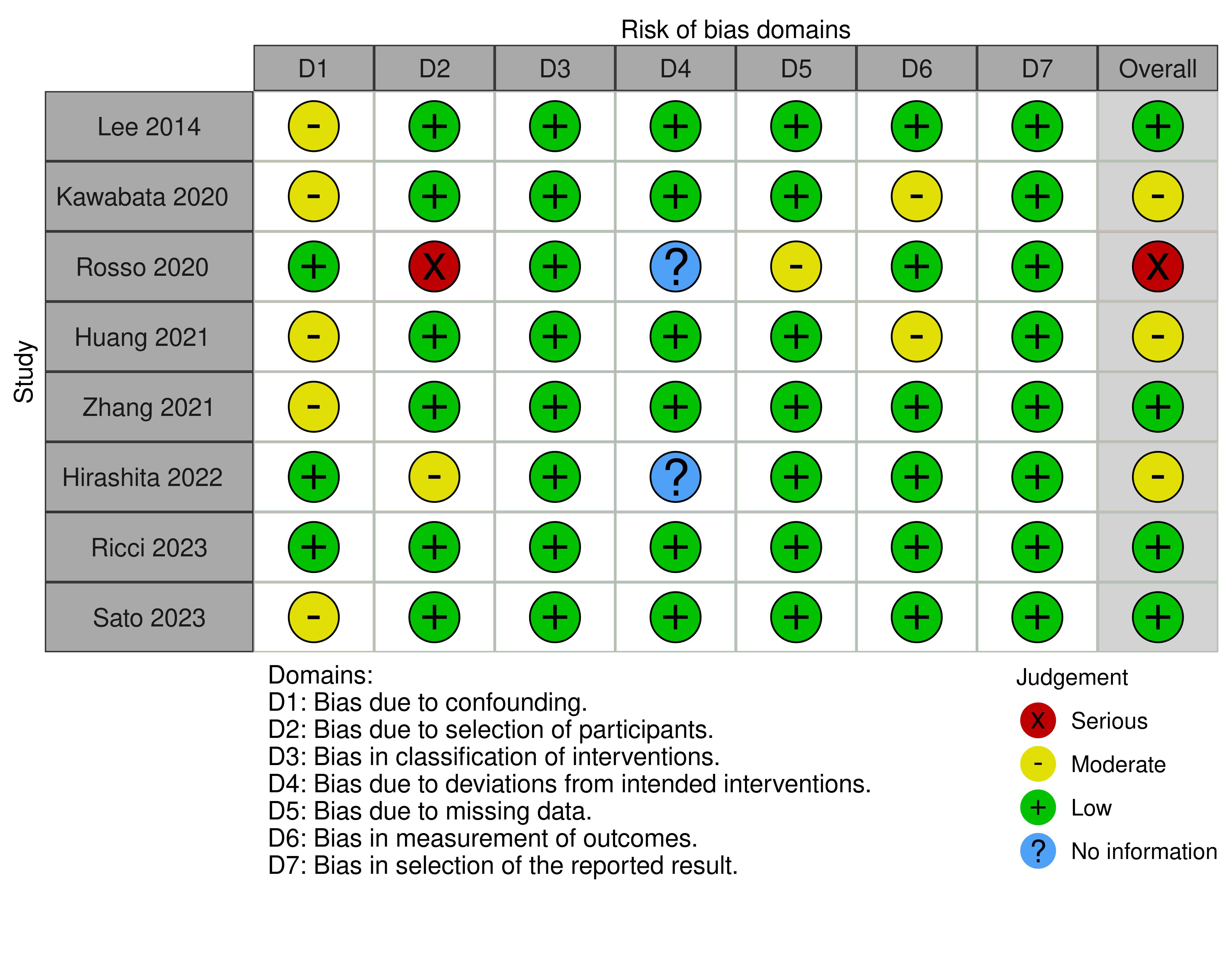

Supplement: Supplementary Figure 1 — Risk of bias assessment across studies. Studies such as Rosso et al., 2020 (26) exhibit a serious risk in D3 and overall bias, while Huang et al., 2021 (22), Zhang et al., 2021 (28), and others show consistently low risk across all domains. Lee et al., 2014 (24) and Kawabata et al., 2020 (23) show moderate risk in D1 and D6, while Hirashita et al., 2022 (21) has a moderate risk in D1 and D2. Two studies (Rosso et al., 2020 (26), Kawabata et al., 2020 (23)) have uncertainties regarding missing data (D5), indicated by blue question marks. The overall bias summary highlights both high- and low-risk studies, providing an overview of the potential limitations in the included literature. [file Image1.jpeg]

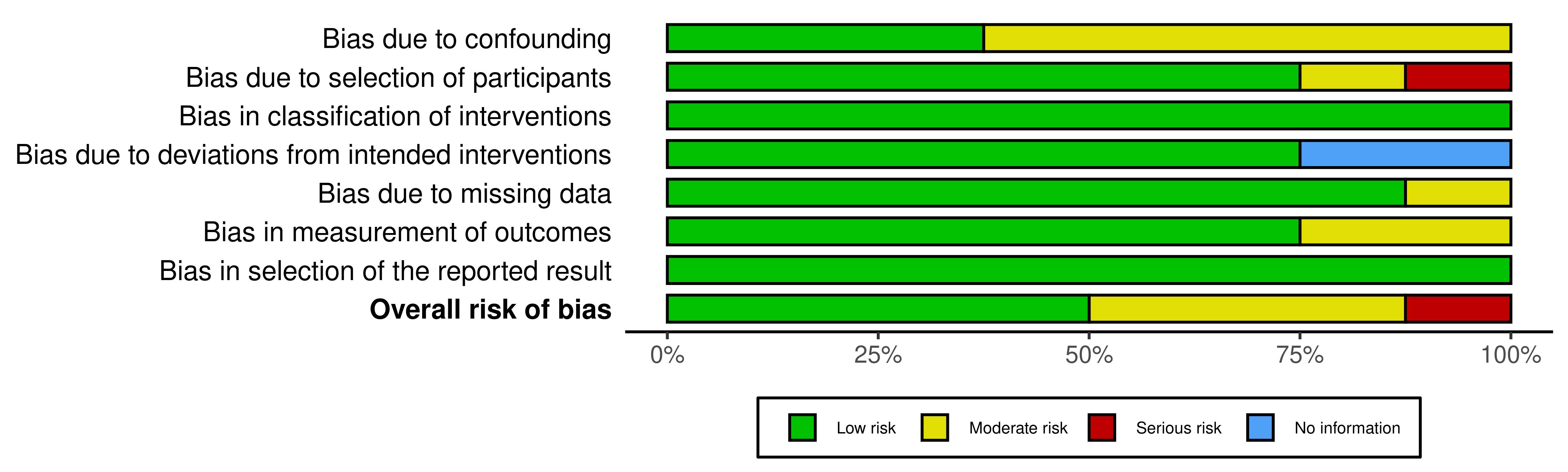

Supplement: Supplementary Figure 2 — Risk of bias summary across bias domains. [file Image2.jpeg]
