# Supplementary material for: A comparative analysis and survival analysis of open versus minimally invasive radical antegrade modular pancreatosplenectomy for pancreatic cancer: a systematic review and meta-analysis
Source: Front Oncol. 2025 Jan 23;14:1513520. doi: 10.3389/fonc.2024.1513520 (PMC11798776; doi:10.3389/fonc.2024.1513520)
Supplement: Supplementary file 3 [file DataSheet1.pdf]

Database	Search Strategy
Embase	radical AND antegrade AND modular AND pancreatosplenectomy
Pubmed	radical antegrade modular pancreatosplenectomy [Title/Abstract]
Cochrane Library	(radical antegrade modular pancreatosplenectomy): ti, ab, kw
