# Supplementary material for: A comparative analysis and survival analysis of open versus minimally invasive radical antegrade modular pancreatosplenectomy for pancreatic cancer: a systematic review and meta-analysis
Source: Front Oncol. 2025 Jan 23;14:1513520. doi: 10.3389/fonc.2024.1513520 (PMC11798776; doi:10.3389/fonc.2024.1513520)
Supplement: Supplementary file 4 [file DataSheet2.pdf]

	Hirashita Lap(n=19)	Hirashita Open(n=31)	Huang Lap(n=20)	Huang Open(n=31)	Kawabata Lap(n=15)	Kawabata Open(n=15)	Lee Lap(n=12)	Lee Open(n=78)	Ricci Lap(n=93)	Ricci Open(n=85)	Rosso Lap(n=17)	Rosso Open(n=6)	Sato Lap(n=43)	Sato Open(n=75)	Zhang Lap(n=25)	Zhang Open(n=23)
Age	73.8±8.1	71.4±9.1	67.2±8.4	66.9±9.1	76(55-86)	74(52-85)	63.3±9.9	51.2±9.9	68.6±8.8	67.6±11.4	median 71	median 75	69(35-84)	70(37-89)	64.72±9.11	63.26±8.74
Male/Female	13/6	20/11	10/10	15/16	9/6	9/6	7/5	47/31	46/47	38/47	8/9	4/2	18/25	49/26	17/8	11/12
BMI(kg/m^2)	23.8±7.4	22.9±3.4	23.4±2.5	22.5±2.0	21.9(13.7-28.5)	22.3(17.1-27.9)	23.9±3.4	22.4±3.1	25.3±4.4	24.4±4.4			21.1(14.6-29.6)	21.8(15.6-30.1)	22.29±2.87	23.54±3.10
History of Laparoscopy	9(47%)	14(31%)														
CEA(ng/ml)	3.7±2.3	4.5±7.5											2.8(0.7-31.6)	2.8(0.7-15.7)		
CA199(U/ml)	102±203	489±1009											27.7(2.0-6743.7)	52.3(2.0-27719.6)		
CA199(>34U/L)															23(92%)	18(78.3%)
ASA																
1			11(55%)	15(48%)	0	1(7%)							6(14%)	20(27%)		
2			8(40%)	15(48%)	13(87%)	11(73%)							36(84%)	39(52%)		
3			1(5%)	1(3%)	2(13%)	3(20%)							1(2%)	6(8%)		
DM					3(20%)	3(20%)			10(11%)	7(8%)	9(53%)	4(67%)	11(25.6%)	24(32.0%)		
Operation Time(min)	397±78	319±80	273.8±90.3	264.3±77.1	423(256-628)	393(280-463)	324.3±154.2	270.1±140.4			NA	median 450	364(250-547)	303(182-579)	212.20±66.31	203.13±39.73
Blood Loss(ml)	299±237	576±78	252.5±198.3	472.6±428.0	220(40-1030)	30(0-100)	445.8±346.1	669.5±776.1					125(5-500)	390(40-1340)	402.00±258.80	506.52±418.41
Blood Transfusion	2(11%)	3(10%)	2(10%)	11(35%)	0	0	2(16.69%)	19(24.4%)					0	1(1.3%)	3(12%)	6(24%)
Anterior/Posterior RAMPS					8/7	13/2			85/8	81/4			26/17	24/51		
Vascular Resection									12(12.9)	17(20)						
Extended resection			6(30%)	11(35%)	4(27%)	3(20%)	1(8%)	27(35%)	78(84%)	71(84%)						
Left adrenal gland			3(15%)	8(26%)												
Stomach			1(5%)	2(6%)												
Colon			2(10%)	1(3%)												
Postoperative Mortality					0	0	0	2(2.6%)					0	0		
Postoperative Morbidity					5(33.3%)	3(20.0%)	3(25%)	29(37.2%)	17.4(17.2%)	13.6(13.2%)	9(53%)	3(50%)			4(16%)	3(13.0%)
Clavien-Dindo classification																
I			15(75%)	18(58%)												
II			4(20%)	12(39%)												
III			1(5%)	1(32%)	1(6.67)	1(6.67)							8(18.6%)	13(17.8%)		
Clavien-Dindo≥lla									19(20.4)	13(15.3)	5(29%)	2(33%)				
POPF											3(18%)	2(33%)			2(8.0%)	0(0.0%)
Grade A			5(25%)	4(13%)	3(20%)	2(13%)	2(16.69%)	10(12.8%)					3(7%)	9(12%)		
Grade B			8(40%)	10(32%)	1(7%)	1(7%)	0	6(7.7%)					16(37%)	20(27%)		
Grade C			1(5%)	0	0	0	0	2(2.6%)					0	0		
POPF(≥B)	2(11%)	6(19%)	9(45%)	14(45%)					38(40.9)	17(20)						
DGE	1(5%)	3(10%)	3(15%)	0			1(8.3%)	3(3.8%)	13(14)	7(8.2)			3(7.0%)	5(6.7%)	2(8.0%)	2(8.7%)
Grade A					1(6.7)	1(7%)										
Grade B					2(13.3)	0										
Grade C					1(6.7)	0										
Peritoneal Infection			2(10%)	2(6%)												
Chyle Leak			2(10%)	4(13%)			0	1(1.3%)								
Thrombocythemia			8(40%)	17(55%)												
Reoperation					0	0					1(6%)	1(6%)				
SSI					0	0							2(4.7%)	10(13.3%)	0(0.0%)	1(4.3%)
PPH					0	0	0	2(2.6%)	6(6.5)	2(2.3)	1(6%)	1(6%)	0	1(1.3%)	1(4%)	0
Intestinal Obstruction							0	2(2.6%)							0(0.0%)	0(0.0%)
Liver Abscess							1(8.3%)	0								
Deep Vein Thrombosis							0	2(2.6%)								
Length of Drainage(day)					5(3-81)	5(3-32)										
Time Between Surgery and AC(day)	36.6±9.5	41.9±27.1			36(9-120)	27(7-58)	37.7±14.4	66.21±70.48					54(26-115)	59.5(33-125)		
Time to Anal Exhaust(days)			2.5±0.8	3.2±1.2												
Time to Oral Intake (days)			2.9±1.0	3.7±1.6	6(4-16	4(2-58)									4.84±1.72	5.61±1.56

	Hirashita Lap(n=19)	Hirashita Open(n=31)	Huang Lap(n=20)	Huang Open(n=31)	Kawabata Lap(n=15)	Kawabata Open(n=15)	Lee Lap(n=12)	Lee Open(n=78)	Ricci Lap(n=93)	Ricci Open(n=85)	Rosso Lap(n=17)	Rosso Open(n=6)	Sato Lap(n=43)	Sato Open(n=75)	Zhang Lap(n=25)	Zhang Open(n=23)
LOS(days)			19.0±9.9	19.6±16.8	16(8-96)	12(8-35)	12.3±6.8	22.4±21.6	13(0.7)	11.9(6.4)					11.72±5.24	12.91±5.02
Postoperative LOS(day)	21.5±10.5	29.4±23.3											17(8-47)	21(10-80)		
Cost in the Hospital(dollar)			8,270±2,590	8,020±1,350												
Adjuvant Therapy	13(68%)	21(68%)	14(70)	25(80%)	12(80%)	11(73.3%)	7(58.3%)	55(70.5%)					39(90.7%)	60(80.0%)	23(92%)	19(83%)
Tumor location at diagnosis																
Body					6	4	8(66.6%)	35(48.9%)					20(47%)	46(61%)		
Body-tail					6	6										
Tail					3	5	4(33.3%)	37(47.4%)					23(53%)	29(39%)		
Size(mm)	27.9±22.5	34.1±18.3	4.2±1.9	4.2±1.6	30.1(1.3-40.7)	35.0(4.3-74.0)	28±13	35±19	29.9±10.9	34±15.6			24(4-78)	30(2-70)	36.7±17.1	43.7+20.3
T stage																
T1	5(26%)	3(10%)	2(10%)	5(16%)	2(14%)	3(21%)	0	5(6.4%)								
T2	1(5%)	1(3%)	8(40%)	9(29%)	2(14%)	1(7%)	0	7(8.9%)								
T3	13(68%)	27(87%)	8(40%)	15(48%)	6(42%)	5(35%)	12(100%)	62(79.5%)								
T4			2(10%)	2(6%)	5(35%)	6(42%)	0	4(5.1%)								
N stage																
N0			14(70%)	19(61%)			9(75%)	41(52.6%)								
N1			6(30%)	10(32%)			3(25%)	37(47.4%)								
N2			0	2(6%)												
R stage																
R0							12(100%)	67(85.9%)								
R1							0	8(10.3%)								
R2							0	3(3.8%)								
TNM stage																
I			7(35%)	9(29%)									18(42%)	28(37%)		
II			9(45%')	16(52)									19(44%)	37(49)		
III			4(20%)	6(19%)									3(7%)	8(11%)		
Retrieved LNs	14±17	19±18	9.6±6.4	12.8±5.8	19(9-71)	22(8-37)	10.5±7.1	13.8±11.1	31.6±15.7	25.7±14.7	31	37	24(8-49)	32(3-83)	15.84±6.71	18.22±7.95
Dissected LNs around the SMA					2(0-15)	5(0-13)										
Peripheral Infiltration																
Serosal Invasion	8(42%)	14(45%)														
Retroperitoneal Invasion	11(58%)	22(71%)														
Plexus Invasion	0	4(13%)													1(4%)	0
Adjacent Organ Invasion	0	0														
Perineural Invasion							5(41.79%)	29(37.2%)							15/10	17/6
Differentiated degree																
Well			1(5%)	1(3%)			2(16.6%)	14(17.9%)							17(68%)	17(74%)
Moderate			8(40%)	9(29%)			8(66.6%)	53(67.9%)								
Poor			11(55%)	21(67%)			2(16.6%)	10(12.8%)								
Undifferentiated							0	1(1.3%)								
positive margin			0	1(3%)	1(7%)	0	0	9(12%)	41(44.1)	36(42.3)	0	0	0	7(9%)	2(8%)	2(9%)
Positive Pancreas Cut-off Margin	1(5%)	1(3%)														
Positive Peripancreatic Tissue Margin	1(5%)	3(10%)														

ASA, American Society of Anesthesiologists score; DM, diabetes mellitus; POPF, postoperative pancreatic fistula; DGE, delayed gastric emptying; SSI, surgical site infection; PPH, post-pancreatectomy hemorrhage; AC, adjuvant chemotherapy; LOS, length of stay; LN, lymph node; SMA, superior mesenteric artery;
